# A Retrospective Analysis of Dabrafenib and/or Dabrafenib Plus Trametinib Combination in Patients with Metastatic Melanoma to Characterize Patients with Long-Term Benefit in the Individual Patient Program (DESCRIBE III)

**DOI:** 10.3390/cancers13102466

**Published:** 2021-05-18

**Authors:** Victoria G. Atkinson, Pietro Quaglino, Massimo Aglietta, Michele Del Vecchio, Roberta Depenni, Francesca Consoli, Dimitrios Bafaloukos, Pier Francesco Ferrucci, Skaiste Tulyte, Ivana Krajsová, Paolo A. Ascierto, Rossana Gueli, Ana Arance, Helen Gogas, Hiya Banerjee, Teddy Saliba, Egbert de Jong, Bart Neyns

**Affiliations:** 1 Division of Cancer Services, Princess Alexandra Hospital, University of Queensland, Brisbane, QLD 4102, Australia; 2 Department of Medical Sciences, Section of Dermatology, University of Turin, 10124 Turin, Italy; pietro.quaglino@unito.it; 3 Department of Oncology, University of Torino, 10124 Turin, Italy; massimo.aglietta@ircc.it; 4 Department of Medical Oncology, Candiolo Cancer Institute, FPO-IRCCS, 10060 Candiolo (Torino), Italy; 5 Unit of Melanoma Medical Oncology, Department of Medical Oncology and Hematology, Fondazione IRCCS Istituto Nazionale dei Tumori, 20133 Milan, Italy; Michele.DelVecchio@istitutotumori.mi.it; 6 Department of Oncology and Hematology, University of Modena and Reggio Emilia, 41121 Modena, Italy; depenni.roberta@policlinico.mo.it; 7 Medical Oncology, ASST Spedali Civili, 25123 Brescia, Italy; francesca.consoli@libero.it; 8 Medical Oncology, Metropolitan Hospital, 18547 Athens, Greece; dimmp@otenet.gr; 9 Tumor Biotherapy Unit, Department of Experimental Oncology, IEO—Istituto Europeo di Oncologia—IRCCS, 20141 Milan, Italy; pier.ferrucci@ieo.it; 10 Hematology, Oncology and Transfusion Medicine Center, Vilnius University, 08410 Vilnius, Lithuania; skaiste.tulyte@santa.lt; 11 Department of Dermatovenerology, University Hospital Prague, Charles University, 12808 Prague, Czech Republic; ivana.krajsova@vfn.cz; 12 Melanoma Unit, Cancer Immunotherapy and Innovative Therapies, Istituto Nazionale Tumori-IRCCS “Fondazione G. Pascale”, 80131 Naples, Italy; paolo.ascierto@gmail.com; 13 Oncology Unit, ASST Sette Laghi, 21100 Varese, Italy; rossana.gueli@asst-settelaghi.it; 14 Department of Medical Oncology, Hospital Clinic of Barcelona, 08036 Barcelona, Spain; amarance@clinic.cat; 15 Department of Internal Medicine, Laiko General Hospital, National and Kapodistrian University of Athens School of Medicine, 11527 Athens, Greece; helgogas@gmail.com; 16 Novartis Pharmaceuticals Corporation, East Hanover, NJ 07936, USA; hiya.banerjee@novartis.com (H.B.); teddy.saliba@novartis.com (T.S.); 17 Novartis AG, 4057 Basel, Switzerland; egbert.de_jong@novartis.com; 18 Medical Oncology, Universitair Ziekenhuis Brussel, 1090 Brussels, Belgium; Bart.Neyns@uzbrussel.be

**Keywords:** *BRAF* V600, chart review, dabrafenib, melanoma, real-world, trametinib

## Abstract

**Simple Summary:**

Compassionate-use programs provide an opportunity to retrospectively evaluate the treatment patterns and clinical outcomes in a real-world setting to validate the results derived from controlled randomized clinical trials. The COMBI-d and COMBI-v studies established the superior efficacy of dabrafenib + trametinib (dab + tram) versus BRAF inhibitor monotherapy in patients with *BRAF* V600–mutant metastatic melanoma. In light of their five-year results demonstrating long-term benefit with first-line dab + tram, it is important to get a real-world perspective of the long-term treatment duration for dab + tram. DESCRIBE III was designed to retrospectively evaluate the impact of patient characteristics on the long-term outcomes of dab + tram in a real-world setting based on the duration of clinical benefit. Consistent with the findings from the pooled analysis of COMBI-d and COMBI-v, lower LDH level and <3 metastatic sites at baseline were associated with a longer duration of treatment benefit in a real-world setting.

**Abstract:**

The dabrafenib plus trametinib (dab + tram) combination has demonstrated durable long-term efficacy in patients with *BRAF* V600–mutant metastatic melanoma. However, real-world data characterizing patients with long-term benefit are limited. DESCRIBE III was a global, observational, retrospective, chart review study in patients with unresectable or metastatic melanoma treated with dab monotherapy and/or dab + tram combination therapy as part of the Named Patient Program or Individual Patient Program. Overall, 509 patients were enrolled. Patients were categorized into three groups based on their observed treatment duration: long-term (on therapy ≥12 months), intermediate (on therapy ≥6 months and <12 months), and short-term (on therapy <6 months) duration of benefit. More patients in the short-term duration of benefit group had baseline characteristics associated with poor prognosis compared with the other two groups. Median lactate dehydrogenase (LDH) levels (368 U/L) at baseline were also higher in the short-term duration of benefit group. No new safety signals were identified. DESCRIBE III identified baseline characteristics associated with long-term benefit of dab + tram. Lower LDH level and <3 metastatic sites at baseline were associated with a longer duration of benefit, confirming that the findings from COMBI-d and COMBI-v are relevant to patients treated in a real-world setting.

## 1. Introduction

The COMBI-d and COMBI-v studies established the superior efficacy of the BRAF inhibitor dabrafenib (dab) in combination with the MEK inhibitor trametinib (tram) versus BRAF inhibitor monotherapy in patients with *BRAF* V600–mutant metastatic melanoma [1,2]. A pooled analysis of the COMBI-d and COMBI-v studies demonstrated the long-term clinical benefit of this combination (dab + tram), reporting a five-year progression-free survival (PFS) rate of 19% and an overall survival (OS) rate of 34% in patients with *BRAF* V600–mutant metastatic melanoma. Analysis of multivariate factors indicated several baseline characteristics associated with improved PFS and OS rates. For example, a subset of patients treated with dab + tram, with a normal lactate dehydrogenase (LDH) level and <3 metastatic sites at baseline, achieved a PFS rate of 31% and an OS rate of 55% at five years [3]. Overall, results from the pooled analysis of both clinical trials suggest that patients with *BRAF* V600–mutant metastatic melanoma having a low initial tumor and disease burden are more likely to achieve long-term benefit from dab + tram therapy. However, data supporting this observation have been limited to clinical trials, and the influence of baseline factors on the outcomes of long-term benefit with dab + tram has not been investigated in a real-world setting.

Analyses from large population-based studies are critical tools in extending and confirming the results derived from controlled randomized clinical trials. Two real-world studies (DESCRIBE I [*N* = 331] and DESCRIBE II [*N* = 271]) evaluated the treatment patterns and clinical outcomes of patients with *BRAF* V600–mutant unresectable or metastatic melanoma treated with either dab monotherapy or dab + tram combination therapy enrolled in the Named Patient Program (NPP). Results from these studies demonstrated that efficacy and safety outcomes were consistent with those observed in randomized clinical studies. However, the impact of baseline characteristics on long-term outcomes was not evaluated in DESCRIBE I and DESCRIBE II [4,5].

DESCRIBE III was designed to retrospectively evaluate the impact of patient characteristics on the long-term outcomes of dab + tram in a real-world setting based on the duration of clinical benefit.

## 2. Materials and Methods

### 2.1. Study Design

DESCRIBE III was a global, observational, retrospective, chart review study conducted in patients with *BRAF* V600–mutant unresectable or metastatic melanoma treated with either dab monotherapy and/or dab + tram combination therapy as part of the NPP or Individual Patient Program (IPP).

Eligible patients were aged ≥18 years with histologically confirmed *BRAF* V600–mutant unresectable or metastatic cutaneous melanoma, had received at least one dose of dab and/or dab + tram as part of the NPP/IPP, and had ≥12 months of extractable chart data after the initiation of dab and/or dab + tram therapy. Patients who did not participate in the NPP/IPP, were part of a dab and/or dab + tram investigational trial, or received dab and/or dab + tram therapy for <12 months at the time of site initiation were excluded from the study.

Retrospective patient data (patient characteristics, clinical disease status, drug dosing, disease progression status, treatment patterns, survival status, and safety data) were taken from the medical charts of all patients from the date of initiation of dab and/or dab + tram therapy until death or the date of study completion (for surviving patients).

In this study, the duration of treatment was used to define treatment benefit and categorize patient groups for a retrospective analysis. Patients were categorized into one of the following three benefit groups based on the observed duration of treatment within the NPP/IPP: long-term duration of benefit group, which included patients who received dab + tram for ≥12 months; intermediate duration of benefit group, which included patients who received dab + tram for ≥6 months and <12 months; and short-term duration of benefit group, which included patients who received dab + tram for <6 months.

### 2.2. Objectives and Assessments

The primary objective was to describe the baseline characteristics (patient demographics and disease characteristics at the time of initiation of dab or dab + tram therapy) for each of the three duration of benefit groups. Secondary objectives were safety during treatment with dab and dab + tram, clinical benefit rate (CBR), PFS, and OS. Other objectives included evaluation of treatment patterns and duration, including time to discontinuation of initial therapy, dose interruptions, and dose adjustments in the initial and subsequent therapies for the three treatment groups.

Safety assessments consisted of recording all adverse events (AEs), serious AEs (SAEs), and AEs of special interest (AESIs) along with their severity and relationship to the study drug. CBR was defined as patients with complete response (CR) or partial response (PR) along with those who achieved stable disease (SD) ≥24 weeks; PFS was defined as time from the initiation of dab and/or dab + tram therapy to the first documented progressive disease (PD) or death due to any cause; and OS was defined as time from the initiation of dab and/or dab + tram therapy to death due to any cause. All efficacy objectives were investigator assessed based on the Response Evaluation Criteria in Solid Tumors (RECIST) version 1.1.

No statistical sample size calculation was performed. Based on the sample sizes of DESCRIBE I and DESCRIBE II, approximately 600 patients were planned to be included in this study with a target of approximately 200 patients per group. Baseline characteristics were summarized by descriptive statistics in each of the three treatment benefit groups separately. All safety and efficacy data were analyzed descriptively. Kaplan–Meier analysis was used to analyze PFS and OS. Best overall response and CBR were analyzed by benefit group and overall by means of a frequency distribution. Two-sided 95% confidence intervals (CIs) were calculated using the Clopper–Pearson method for the success rates.

### 2.3. Approvals by Ethics Committee or Institutional Review Boards

The study protocol was approved by an independent ethics committee or institutional review board in compliance with the local country and regulatory guidelines. This study was conducted in accordance with the International Conference on Harmonization Good Clinical Practice guidelines as applicable to observational research, patient privacy requirements, and ethical principles outlined in the Declaration of Helsinki.

## 3. Results

### 3.1. Patients and Characteristics

**Figure 1:**
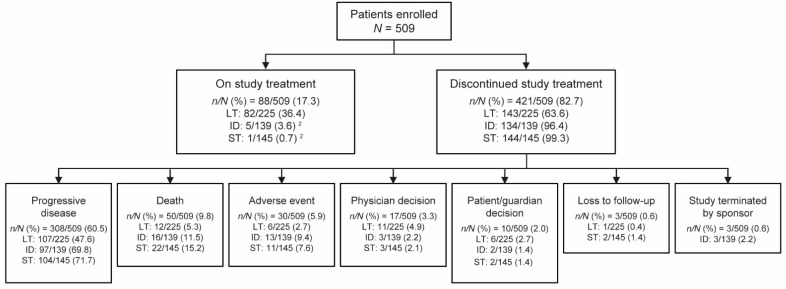
Patient disposition ^1^. ^1^ Intention-to-treat population. ^2^ Patients who were undergoing treatment at the time of site initiation for <12 months were (erroneously) enrolled in the study. ID, intermediate duration of benefit; LT, long-term duration of benefit; ST, short-term duration of benefit.

In this study, data for 509 enrolled patients were analyzed. Based on their observed treatment duration, 225 patients were categorized into the long-term duration of benefit group, 139 into the intermediate duration of benefit group, and 145 into the short-term duration of benefit group. At data cutoff (14 December 2018), 88 patients (17.3%) were on treatment, while 421 patients (82.7%) discontinued treatment (Figure 1). The most common reason for discontinuation across all three groups was PD (*n* of *N* = 308 of 509; 60.5%). Discontinuations in the short-term and intermediate duration of benefit groups were mostly due to PD (short-term, 104 of 145 (71.7%); intermediate duration, 97 of 139 (69.8%)), followed by death (short-term, 22 of 145 (15.2%); intermediate duration, 16 of 139 (11.5%)), and AEs (short-term, 11 of 145 (7.6%); intermediate duration, 13 of 139 (9.4%)). In the long-term duration of benefit group, there were notably lower discontinuations due to PD (107 of 225 (47.6%)), lower death rate (12 of 225 (5.3%)), and very low discontinuations due to AEs (6 of 225 (2.7%)).

Demographics and baseline characteristics are outlined in Table 1. Several baseline characteristics such as age, sex, and race were similar across all duration of benefit groups. The BRAF V600 mutation subtypes were not substantially different between the groups. A higher proportion of patients in the short-term duration of benefit group had less favorable characteristics at baseline compared with the long-term duration of benefit group: Eastern Cooperative Oncology Group performance status (ECOG PS) ≥1 (29.0% versus 16.0%), stage III/IV disease at initial diagnosis of melanoma (69.0% versus 57.8%), and ≥3 metastatic sites (33.8% versus 20.9%). Median LDH levels at baseline were higher in the short-term duration of benefit group compared with the long-term duration of benefit group (368 U/L versus 277 U/L). Brain metastases were somewhat similar across all the groups (short-term, 22.1%; intermediate duration, 18.7%; long-term, 17.3%); however, liver metastases were more common in the short-term duration of benefit group than in the long-term duration of benefit group (28.3% versus 11.1%).

Overall, patients in the short-term duration of benefit group received more lines of prior therapies, including systemic therapy, surgery, and radiotherapy. A higher proportion of patients in the short-term duration of benefit group received ≥2 prior antineoplastic therapies than in the long-term duration of benefit group (21.4% versus 9.8%). More patients in the short-term duration of benefit group had PD compared with the long-term duration of benefit group (32.3% versus 16.9%). Patients in the long-term duration of benefit group had a longer median duration (5.0 months) of the last treatment regimen prior to study treatment and longer median duration (13.0 months) of best response to prior treatment than those in the short-term duration of benefit group (Table 2).

### 3.2. Safety

Overall, 339 patients (66.6%) had ≥1 AE; 96 patients (18.9%) had a grade ≥3 AE. The overall incidence of AEs was slightly higher in the long-term duration of benefit group than in the short-term duration of benefit group (72.0% versus 62.1%). The rate of dose adjustments/interruptions due to AEs was also slightly higher in the long-term duration of benefit group than in the short-term duration of benefit group (34.2% versus 20.7%). However, the rate of discontinuations due to AEs was lower in the long-term duration of benefit group than in the short-term duration of benefit group (4.9% versus 7.6%; Table 3).

The most common AE was pyrexia (*n* = 133 (26.1%); short-term, 22.1%; long-term, 28.4%). The other frequent AEs were rash (*n* = 47 (9.2%)), asthenia (*n* = 45 (8.8%)), fatigue (*n* = 34 (6.7%)), nausea (*n* = 34 (6.7%)), diarrhea (*n* = 32 (6.3%)), and hyperkeratosis (*n* = 31 (6.1%)). Overall, 75 patients (14.7%) had an SAE, of whom 11 (2.2%) had a grade 5 SAE. The most common SAEs were pyrexia (2.0%) and headache (1.0%), which were more frequent in the long-term duration of benefit group. Overall, 267 patients (52.5%) had ≥1 treatment-related AE. Forty-seven patients (9.2%) experienced grade ≥3 AEs (Table 3). Of these, 43 were grade 3, three were grade 4 (diarrhea, ventricular fibrillation, and hyponatremia), and one was a grade 5 event (pulmonary hypertension). Pulmonary hypertension (grade 5) led to treatment discontinuation and death of the patient. The patient also had a prior history of hypertension, ventricular septal defect, and diabetes. There was inadequate information to determine if there was any relationship between the treatment and worsening of pulmonary hypertension leading to the death.

There were 127 deaths (25.0%) during the study, of which 105 were due to progressing metastatic melanoma. There was a greater number of on-treatment deaths in the short-term duration of benefit group (*n* = 56 (38.6%)) versus the long-term duration of benefit group (*n* = 32 (14.2%); Table S1).

### 3.3. Treatment Patterns

Overall, 472 patients (92.7%) were treated with dab + tram combination therapy and 37 (7.3%) with dab monotherapy. Patients received a median average daily dose of dab 300 mg/day (range, 83–600 mg/day) across all groups, and patients who received the combination treatment with tram had received a median average daily dose of tram 2.0 mg/day (range, 0–3 mg/day) across all groups. Most dose reductions (dab, 15.1%; tram, 8.4%) and treatment interruptions (dab, 28.9%; tram, 25.3%) occurred in the long-term duration of benefit group. The rate of permanent discontinuation of treatment was higher in the intermediate and short-term duration of benefit groups (Table 4). The main reason for the permanent discontinuation of treatment was PD, whereas the main reason for dose reduction and treatment interruption was AEs (Table S2).

### 3.4. Efficacy

Given that patients were grouped based on the duration of benefit, overall, the efficacy outcomes were expected to be better in the long-term duration of benefit group compared with the other two groups. Consistent with this, the CBR was higher in the long-term duration of benefit group (81.3%) versus the short-term duration of benefit group (9.7%; Table 5). Similarly, fewer patients in the long-term duration of benefit group had a best response of PD compared with the short-term duration of benefit group (8.0% versus 60.7%). Median PFS and OS were also longest in the long-term duration of benefit group (Figure S1A,B).

## 4. Discussion

Compassionate-use programs (NPP/IPP) provide an opportunity to retrospectively evaluate the treatment patterns and clinical outcomes in a real-world setting to validate the results derived from controlled randomized clinical trials. Two real-world studies (DESCRIBE I and DESCRIBE II) examined outcomes of the therapies evaluated in the BREAK (dab monotherapy) and COMBI-d and COMBI-v (dab + tram combination therapy) studies and demonstrated consistency with these previous pivotal clinical studies [4,5]. However, DESCRIBE I and DESCRIBE II had limited follow-up and did not evaluate the impact of baseline characteristics on long-term outcomes; thus, a real-world analysis with longer follow-up was needed. In light of the five-year COMBI-d and COMBI-v data demonstrating long-term benefit with first-line dab + tram, it is important to get a real-world perspective of the long-term treatment duration for dab + tram [3].

Overall, DESCRIBE III demonstrated that patients in the short-term duration of benefit group had a more aggressive disease compared with those in the long-term duration of benefit group. The short-term duration of benefit group had a higher percentage of patients with ECOG PS ≥1, advanced disease, and elevated disease burden. These observations are consistent with those from clinical trials of patients with metastatic *BRAF* V600–mutant melanoma who received targeted therapy [3,6,7].

Serum LDH levels and the number of metastatic sites at baseline are indicators of poor prognosis in cancer patients [8]. In the registrational studies of dab + tram (COMBI-d/COMBI-v), patients with normal LDH level and <3 metastatic sites had the longest PFS and OS, whereas patients with LDH level ≥2 times the upper limit of normal had the shortest PFS and OS [7,9]. In the five-year pooled analysis of COMBI-d/COMBI-v, patients with normal LDH level and <3 metastatic sites at baseline were identified as the most favorable subgroup with an OS of 55% and a PFS of 31% [3]. This was consistent with the current study where the median LDH levels at baseline were the highest in the short-term duration of benefit group at 368 U/L, followed by the intermediate (303 U/L) and long-term (277 U/L) duration of benefit groups. Furthermore, 79.1% of patients in the long-term duration of benefit group had <3 metastatic sites, compared with 65.5% and 66.2% of patients in the intermediate and short-term duration of benefit groups, respectively. These results demonstrate the impact of patient baseline characteristics on the treatment duration, and therefore, the clinical benefit observed in a controlled clinical trial setting was reiterated in the real-world setting.

Brain metastases are more commonly associated with poor prognosis in melanoma [10,11,12,13]. A notable observation from this analysis was that brain metastases were somewhat similar across all groups (short-term, 22.1%; long-term, 17.3%), while liver metastases were the highest in the short-term duration of benefit group (short-term, 28.3%; long-term, 11.1%). Further characterization of the patients with brain and liver metastases evaluated in this study may be needed to understand these differences.

The safety data were consistent with those seen in prior phase 2 and phase 3 clinical studies as well as in DESCRIBE I and DESCRIBE II [4,5,14,15,16]. Although AEs tend to be under-reported when studies are retrospective in nature, nearly all AEs in DESCRIBE III were grade 1–3, and the most common AEs were pyrexia, rash, asthenia, fatigue, nausea, diarrhea, and hyperkeratosis, similar to those observed in the clinical trial setting [1,2]. The proportion of patients with AEs and some select AEs (e.g., pyrexia) was lower than that reported in phase 3 clinical trials, which may be at least in part due to underreporting of AEs in this retrospective, real-world analysis.

Overall, the AEs were slightly higher in the long-term duration of benefit group (long-term, 72.0%; short-term, 62.1%); however, this was expected as the median duration of exposure to study treatment was much longer in this group. Notably, the rate of dose adjustments/interruptions due to AEs was also slightly higher in the long-term duration of benefit group (34.2% versus 20.7% in short-term). However, the rate of discontinuations due to AEs was lowest in the long-term duration of benefit group (4.9% versus 7.6% in short-term). The frequency of pyrexia (*n* = 133 (26.1%)) was slightly higher in the long-term duration of benefit group (long-term, 28.4%; short-term, 22.1%). An analysis of patients treated with dab + tram across clinical trials showed that AEs from targeted therapy, particularly pyrexia, occurred early after initiation and resolved with time [17]. These observations show that AEs tend to occur early in the treatment course and patients can be managed using established AE management protocols and receive long-term therapy with dose adjustments, which is consistent with dab + tram clinical trial observations [17,18,19].

As this was a retrospective observational study, one must consider the potential limitations. First, patients selected for study inclusion represent a ‘convenience’ sample, in that the records were obtained from physicians and study sites that were willing to participate in the study. Second, the information captured in the electronic case report form (eCRF) was limited to that available in patient medical records held by the physicians participating in the study. Data on health care services received outside the physician’s care setting that were not recorded in the medical chart were not available for this study. Third, as the data were entered into the eCRF directly by the treating physicians (or nurses), there was also a possibility of data errors in the eCRFs. Fourth, since response assessments were not necessarily done on a uniform schedule, any findings regarding the endpoints of clinical response may not be directly comparable. Finally, each treatment duration of benefit group includes patients who discontinued due to reasons other than disease progression, which is reflective of treatment in a real-world setting. However, these limitations are typical of retrospective medical record reviews and are to be expected but did not influence the overall findings of this study.

## 5. Conclusions

In conclusion, DESCRIBE III identified baseline characteristics associated with the long-term treatment benefit of dab + tram. Lower LDH level and <3 metastatic sites at baseline were associated with a longer duration of treatment benefit, consistent with the findings from a pooled analysis of COMBI-d and COMBI-v. Further studies can seek to identify other factors predicting long-term responses in a real-world setting. Additional translational and biomarker research would help further characterize patients who might demonstrate a long-term treatment benefit. DESCRIBE III provides robust real-world data showing the relationship between baseline characteristics, duration of clinical benefit, and safety outcomes in patients treated with dab with or without tram. These data confirm that the findings from COMBI-d and COMBI-v are relevant to patients treated in a real-world setting.

## Figures and Tables

**Table 1:** Baseline and disease characteristics of patients in the benefit groups.

Parameters	Long-Term Duration of Benefit, ≥12 Months (*n* = 225)	Intermediate Duration of Benefit, ≥6 mo and <12 Months (*n* = 139)	Short-Term Duration of Benefit, <6 Months (*n* = 145)	Overall (*N* = 509)
Age, median (range), years ^1^	57 (24–84)	55 (19–83)	53 (18–89)	56 (18–89)
18–65, *n* (%)	159 (70.7)	101 (72.7)	110 (75.9)	370 (72.7)
66–75, *n* (%)	47 (20.9)	29 (20.9)	27 (18.6)	103 (20.2)
≥76, *n* (%)	17 (7.6)	9 (6.5)	8 (5.5)	34 (6.7)
Missing, *n* (%)	2 (0.9)	0	0	2 (0.4)
Sex, *n* (%)				
Male	123 (54.7)	79 (56.8)	87 (60.0)	289 (56.8)
Female	102 (45.3)	60 (43.2)	58 (40.0)	220 (43.2)
Race, *n* (%)				
Caucasian	213 (94.7)	130 (93.5)	141 (97.2)	484 (95.1)
Unknown	11 (4.9)	8 (5.8)	4 (2.8)	23 (4.5)
Other	1 (0.4)	1 (0.7)	0	2 (0.4)
ECOG PS, *n* (%)				
0	131 (58.2)	74 (53.2)	62 (42.8)	267 (52.5)
1	34 (15.1)	27 (19.4)	31 (21.4)	92 (18.1)
≥2	2 (0.9)	5 (3.6)	11 (7.6)	18 (3.5)
Not assessed	58 (25.8)	33 (23.7)	41 (28.3)	132 (25.9)
BRAF mutation status, *n* (%)				
V600E	179 (79.6)	119 (85.6)	105 (72.4)	403 (79.2)
V600K V600G V600R	17 (7.6) 7 (3.1) 1 (0.4)	4 (2.9) 1 (0.7) 0	14 (9.7) 2 (1.4) 0	35 (6.9) 10 (2.0) 1 (0.2)
Other *BRAF* mutations ^2^	21 (9.3)	15 (10.8)	23 (15.9)	59 (11.6)
Missing	0	0	1 (0.7)	1 (0.2)
Diagnosis of disease, *n* (%)				
Cutaneous melanoma	222 (98.7)	136 (97.8)	143 (98.6)	501 (98.4)
Noncutaneous melanoma	3 (1.3)	1 (0.7)	0	4 (0.8)
Missing	0	2 (1.4)	2 (1.4)	4 (0.8)
AJCC 7 stage at initial diagnosis, *n* (%)				
Stage 0	0	1 (0.7)	0	1 (0.2)
Stage I	39 (17.3)	16 (11.5)	12 (8.3)	67 (13.2)
Stage II	49 (21.8)	31 (22.3)	30 (20.7)	110 (21.6)
Stage III	76 (33.8)	44 (31.7)	47 (32.4)	167 (32.8)
Stage IV	54 (24.0)	39 (28.1)	53 (36.6)	146 (28.7)
Unknown/missing	7 (3.1)	8 (5.8)	3 (2.1)	18 (3.5)
Metastatic sites, *n* (%)				
0 ^3^	73 (32.4)	44 (31.7)	37 (25.5)	154 (30.3)
1	56 (24.9)	27 (19.4)	33 (22.8)	116 (22.8)
2	49 (21.8)	20 (14.4)	26 (17.9)	95 (18.7)
3	25 (11.1)	19 (13.7)	16 (11.0)	60 (11.8)
≥4	22 (9.8)	29 (20.9)	33 (22.8)	84 (16.5)
Site of metastasis, *n* (%) ^4^				
Lymph nodes Lung	102 (45.3) 48 (21.3)	72 (51.8) 41 (29.5)	72 (49.7) 43 (29.7)	246 (48.3) 132 (25.9)
Brain	39 (17.3)	26 (18.7)	32 (22.1)	97 (19.1)
Liver	25 (11.1)	29 (20.9)	41 (28.3)	95 (18.7)
Bone	22 (9.8)	22 (15.8)	23 (15.9)	67 (13.2)
Skin	28 (12.4)	11 (7.9)	19 (13.1)	58 (11.4)
Time since initial diagnosis, median (range), mo	27.2 (1–457)	24.7 (0–313)	19.9 (0–275)	24.3 (0–457)
LDH at baseline, median (range), U/L	277.0 (2–3190)	303.0 (3–6811)	368.0 (3–4471)	307.5 (2–6811)

Abbreviations: AJCC, American Joint Committee on Cancer; dab, dabrafenib; ECOG PS, Eastern Cooperative Oncology Group performance status; LDH, lactate dehydrogenase; tram, trametinib. ^1^ Age at initiation of dab monotherapy and/or dab + tram combination therapy. ^2^ Includes tumors with V600 mutation detected but not specified. ^3^ Patients without metastatic sites had unresectable disease. ^4^ Occurring in >10% of patients.

**Table 2:** Previous treatments.

Treatments	Long-Term Duration of Benefit, ≥12 Months (*n* = 225)	Intermediate Duration of Benefit, ≥6 mo and <12 Months (*n* = 139)	Short-Term Duration of Benefit, <6 Months (*n* = 145)	Overall (*N* = 509)
Prior antineoplastic therapies, *n* (%)				
0	154 (68.4)	83 (59.7)	80 (55.2)	317 (62.3)
1	49 (21.8)	31 (22.3)	34 (23.4)	114 (22.4)
2	15 (6.7)	10 (7.2)	19 (13.1)	44 (8.6)
≥3	7 (3.1)	15 (10.8)	12 (8.3)	34 (6.7)
Previous radiotherapies, *n* (%)				
0	195 (86.7)	109 (78.4)	115 (79.3)	419 (82.3)
1–3	30 (13.3)	30 (21.6)	30 (20.7)	90 (17.7)
Prior surgeries, *n* (%)				
0	56 (24.9)	35 (25.2)	20 (13.8)	111 (21.8)
1–2	96 (42.7)	50 (36.0)	53 (36.6)	199 (39.1)
≥3	73 (32.4)	54 (38.8)	72 (49.7)	199 (39.1)
Treatment duration of the last regimen prior to study medication, median (range), mo ^1^	5.0 (0–43)	3.7 (0–38)	2.3 (0–80)	3.2 (0–80)
Best response to treatment prior to study medication, *n* (%) ^1^				
CR	4 (5.6)	3 (5.4)	6 (9.2)	13 (6.8)
PR	10 (14.1)	9 (16.1)	12 (18.5)	31 (16.1)
SD	13 (18.3)	12 (21.4)	8 (12.3)	33 (17.2)
PD	12 (16.9)	11 (19.6)	21 (32.3)	44 (22.9)
Non-CR/non-PD	0	2 (3.6)	0	2 (1.0)
Unknown	32 (45.1)	19 (33.9)	18 (27.7)	69 (35.9)
Duration of best response to the last regimen prior to study treatment, median (range), mo ^1^	13.0 (1–132)	4.0 (1–32)	3.0 (0–32)	5.0 (0–132)

Abbreviations: CR, complete response; PD, progressive disease; PR, partial response; SD, stable disease.^1^ Excluding patients who received study treatment as first-line treatment. Based on the Response Evaluation Criteria in Solid Tumors version 1.1 as documented in the medical records.

**Table 3:** Summary of safety outcomes by group.

AEs, *n* (%)	Long-Term Duration of Benefit, ≥12 Months (*n* = 225)	Intermediate Duration of Benefit, ≥6 mo and <12 Months (*n* = 139)	Short-Term Duration of Benefit, <6 Months (*n* = 145)	Overall (*N* = 509)
Any-grade AEs				
All causality treatment related	162 (72.0)/137 (60.9)	87 (62.6)/70 (50.4)	90 (62.1)/60 (41.4)	339 (66.6)/267 (52.5)
Grade ≥3 AEs				
All causality/ treatment related	46 (20.4)/27 (12.0)	30 (21.6)/11 (7.9)	20 (13.8)/9 (6.2)	96 (18.9)/47 (9.2)
Serious AEs				
All causality/ treatment related	35 (15.6)/13 (5.8)	24 (17.3)/3 (2.2)	16 (11.0)/2 (1.4)	75 (14.7)/18 (3.5)
Grade 5 serious AEs				
All causality/ treatment related	3 (1.3)/0	4 (2.9)/1 (0.7)	4 (2.8)/0	11 (2.2)/1 (0.2)
AEs leading to discontinuation				
All causality/ treatment related	11 (4.9)/7 (3.1)	11 (7.9)/7 (5.0)	11 (7.6)/6 (4.1)	33 (6.5)/20 (3.9)
AEs leading to dose adjustment/ interruption	77 (34.2)	36 (25.9)	30 (20.7)	143 (28.1)
AEs requiring additional therapy	107 (47.6)	52 (37.4)	54 (37.2)	213 (41.8)
Any-grade AE in ≥5% of patients				
Pyrexia	64 (28.4)	37 (26.6)	32 (22.1)	133 (26.1)
Rash	23 (10.2)	17 (12.2)	7 (4.8)	47 (9.2)
Asthenia	24 (10.7)	10 (7.2)	11 (7.6)	45 (8.8)
Fatigue	16 (7.1)	12 (8.6)	6 (4.1)	34 (6.7)
Nausea	13 (5.8)	9 (6.5)	12 (8.3)	34 (6.7)
Diarrhea	18 (8.0)	9 (6.5)	5 (3.4)	32 (6.3)
Hyperkeratosis	12 (5.3)	13 (9.4)	6 (4.1)	31 (6.1)
Arthralgia	18 (8.0)	7 (5.0)	4 (2.8)	29 (5.7)
Headache	17 (7.6)	4 (2.9)	7 (4.8)	28 (5.5)
Any-grade serious AE in ≥1% of patients				
Pyrexia	8 (3.6)	0	2 (1.4)	10 (2.0)
Headache	3 (1.3)	1 (0.7)	1 (0.7)	5 (1.0)
Other AESI in ≥2% of patients				
Neutropenia	13 (5.8)	2 (1.4)	3 (2.1)	18 (3.5)
Peripheral edema	7 (3.1)	1 (0.7)	5 (3.4)	13 (2.6)
Alopecia	7 (3.1)	5 (3.6)	0	12 (2.4)
Pruritus	7 (3.1)	3 (2.2)	2 (1.4)	12 (2.4)
Cough	6 (2.7)	3 (2.2)	1 (0.7)	10 (2.0)

Abbreviations: AE, adverse event; AESI, adverse events of special interest.

**Table 4:** Summary of study treatment.

Study Treatment	Long-Term Duration of Benefit, ≥12 Months (*n* = 225)	Intermediate Duration of Benefit, ≥6 mo and <12 Months (*n* = 139)	Short-Term Duration of Benefit, <6 Months (*n* = 145)	Overall (*N* = 509)
Type of study treatment, *n* (%)				
Dab + tram	216 (96.0)	131 (94.2)	125 (86.2)	472 (92.7)
Dab monotherapy	9 (4.0)	8 (5.8)	20 (13.8)	37 (7.3)
Duration of exposure to any study treatment, median (range), weeks	93.9 (52–275)	37.9 (26–52)	16.1 (2–26)	46.6 (2–275)
Average dose of dab, median (range), mg/day	300 (130–300)	300 (150–600)	300 (83–300)	300 (83–600)
Reduced dose of dab, *n* (%)	34 (15.1)	12 (8.6)	9 (6.2)	55 (10.8)
1–2 dose reductions	32 (14.2)	11 (7.9)	9 (6.2)	52 (10.2)
>2 dose reductions	2 (0.9)	1 (0.7)	0	3 (0.6)
Interrupted dab treatment, *n* (%)	65 (28.9)	35 (25.2)	34 (23.4)	134 (26.3)
1–2 interruptions	46 (20.4)	31 (22.3)	32 (22.1)	109 (21.4)
>2 interruptions	19 (8.4)	4 (2.9)	2 (1.4)	25 (4.9)
Permanent discontinuation of dab, *n* (%)	142 (63.1)	134 (96.4)	144 (99.3)	420 (82.5)
Average dose of tram, median (range), mg/day	2.0 (1–2)	2.0 (0–2)	2.0 (0–3)	2.0 (0–3)
Reduced dose of tram, *n* (%)	19 (8.4)	10 (7.2)	2 (1.4)	31 (6.1)
1 dose reduction	13 (5.8)	8 (5.8)	2 (1.4)	23 (4.5)
2 dose reductions	6 (2.7)	2 (1.4)	0	8 (1.6)
Interrupted tram treatment, *n* (%)	57 (25.3)	31 (22.3)	25 (17.2)	113 (22.2)
1–2 interruptions	40 (17.8)	26 (18.7)	22 (15.2)	88 (17.3)
>2 interruptions	17 (7.6)	5 (3.6)	3 (2.1)	25 (4.9)
Permanent discontinuation of tram, *n* (%)	135 (60.0)	125 (89.9)	124 (85.5)	384 (75.4)

Abbreviations: dab, dabrafenib; tram, trametinib.

**Table 5:** Best overall response rate and clinical benefit rate.

Parameter	Long-Term Duration of Benefit, ≥12 Months (*n* = 225)	Intermediate Duration of Benefit, ≥6 mo and <12 Months (*n* = 139)	Short-Term Duration of Benefit, <6 Months (*n* = 145)	Overall (*N* = 509)
Best overall response, *n* (%)				
CR	67 (29.8)	9 (6.5)	1 (0.7)	77 (15.1)
PR	72 (32.0)	51 (36.7)	13 (9.0)	136 (26.7)
SD	34 (15.1)	14 (10.1)	0	48 (9.4)
PD	18 (8.0)	51 (36.7)	88 (60.7)	157 (30.8)
Non-CR/Non-PD	10 (4.4)	2 (1.4)	0	12 (2.4)
Unknown	24 (10.7)	12 (8.6)	43 (29.7)	79 (15.5)
Clinical benefit rate (CR + PR + non-CR/non-PD + SD > 24 weeks), n (%) [95% CI]	183 (81.3) [75.6–86.2]	76 (54.7) [46.0–63.1]	14 (9.7) [5.4–15.7]	273 (53.6) [49.2–58.0]

Abbreviations: CI, confidence interval; CR, complete response; PD, progressive disease; PR, partial response; SD, stable disease.

## Data Availability

Not applicable.

## Supplementary Materials

The following are available online at https://www.mdpi.com/article/10.3390/cancers13102466/s1, Figure S1: (A) PFS and (B) OS in patients in the long-term, intermediate, and short-term duration of benefit groups. Table S1. On-treatment deaths. Table S2. Reasons for dose reductions, interruptions, and permanent discontinuations.
