# Supplementary material for: A Retrospective Analysis of Dabrafenib and/or Dabrafenib Plus Trametinib Combination in Patients with Metastatic Melanoma to Characterize Patients with Long-Term Benefit in the Individual Patient Program (DESCRIBE III)
Source: Cancers (Basel). 2021 May 18;13(10):2466. doi: 10.3390/cancers13102466 (PMC8158680; doi:10.3390/cancers13102466)
Supplement: Supplementary file 1 [file cancers-13-02466-s001.zip › cancers-1158901 supplementary.pdf]

*cancers*

*Article*

# A Retrospective Analysis of Dabrafenib and/or Dabrafenib Plus Trametinib Combination in Patients with Metastatic Melanoma to Characterize Patients with Long-Term Benefit in the Individual Patient Program (DESCRIBE III)

**Victoria G. Atkinson [1,*], Pietro Quaglino [2], Massimo Aglietta [3], Michele Del Vecchio [4], Roberta Depenni [5], Francesca Consoli [6], Dimitrios Bafaloukos [7], Pier Francesco Ferrucci [8], Skaiste Tulyte [9], Ivana Krajsová [10], Paolo A. Ascierto [11], Rossana Gueli [12], Ana Arance [13], Helen Gogas [14], Hiya Banerjee [15], Teddy Saliba [15], Egbert de Jong [16], and Bart Neyns [17]**

1 Division of Cancer Services, Princess Alexandra Hospital, University of Queensland, Brisbane QLD 4102, Australia
2 Department of Medical Sciences, Section of Dermatology, University of Turin, 10124 Turin, Italy; pietro.quaglino@unito.it
3 Candiolo Cancer Institute FPO-IRCCS, Candiolo, Department of Oncology, University of Torino, 10124 Turin, Italy; massimo.aglietta@ircc.it
4 Unit of Melanoma Medical Oncology, Department of Medical Oncology and Hematology, Fondazione IRCCS Istituto Nazionale dei Tumori, 20133 Milan, Italy; Michele.DelVecchio@istitutotumori.mi.it
5 Department of Oncology and Hematology, University of Modena and Reggio Emilia, 41121 Modena, Italy; depenni.roberta@policlinico.mo.it
6 Medical Oncology, ASST Spedali Civili, 25123 Brescia, Italy; francesca.consoli@libero.it
7 Medical Oncology, Metropolitan Hospital, 18547 Athens, Greece; dimmp@otenet.gr
8 Tumor Biotherapy Unit, Department of Experimental Oncology, IEO—Istituto Europeo di Oncologia—IRCCS, 20141 Milan, Italy; pier.ferrucci@ieo.it
9 Hematology, Oncology and Transfusion Medicine Center, Vilnius University, 08410 Vilnius, Lithuania; skaiste.tulyte@santa.lt
10 Department of Dermatovenerology, University Hospital Prague, Charles University, 12808 Prague, Czech Republic; ivana.krajsova@vfn.cz
11 Melanoma Unit, Cancer Immunotherapy and Innovative Therapies, Istituto Nazionale Tumori-IRCCS "Fondazione G. Pascale" 80131 Naples, Italy; paolo.ascierto@gmail.com
12 Oncology Unit, ASST Sette Laghi, 21100 Varese, Italy; rossana.gueli@asst-settelaghi.it
13 Department of Medical Oncology, Hospital Clinic of Barcelona, 08036 Barcelona, Spain; amarance@clinic.cat
14 Department of Internal Medicine, Laiko General Hospital, National and Kapodistrian University of Athens School of Medicine, 11527 Athens, Greece; helgogas@gmail.com
15 Novartis Pharmaceuticals Corporation, East Hanover, NJ 07936, USA; hiya.banerjee@novartis.com (H.B.); teddy.saliba@novartis.com (T.S.)
16 Novartis AG, 4057 Basel, Switzerland; egbert.de_jong@novartis.com
17 Medical Oncology, Universitair Ziekenhuis Brussel, 1090 Brussels, Belgium; Bart.Neyns@uzbrussel.be
* Correspondence: victoria.atkinson@health.qld.gov.au

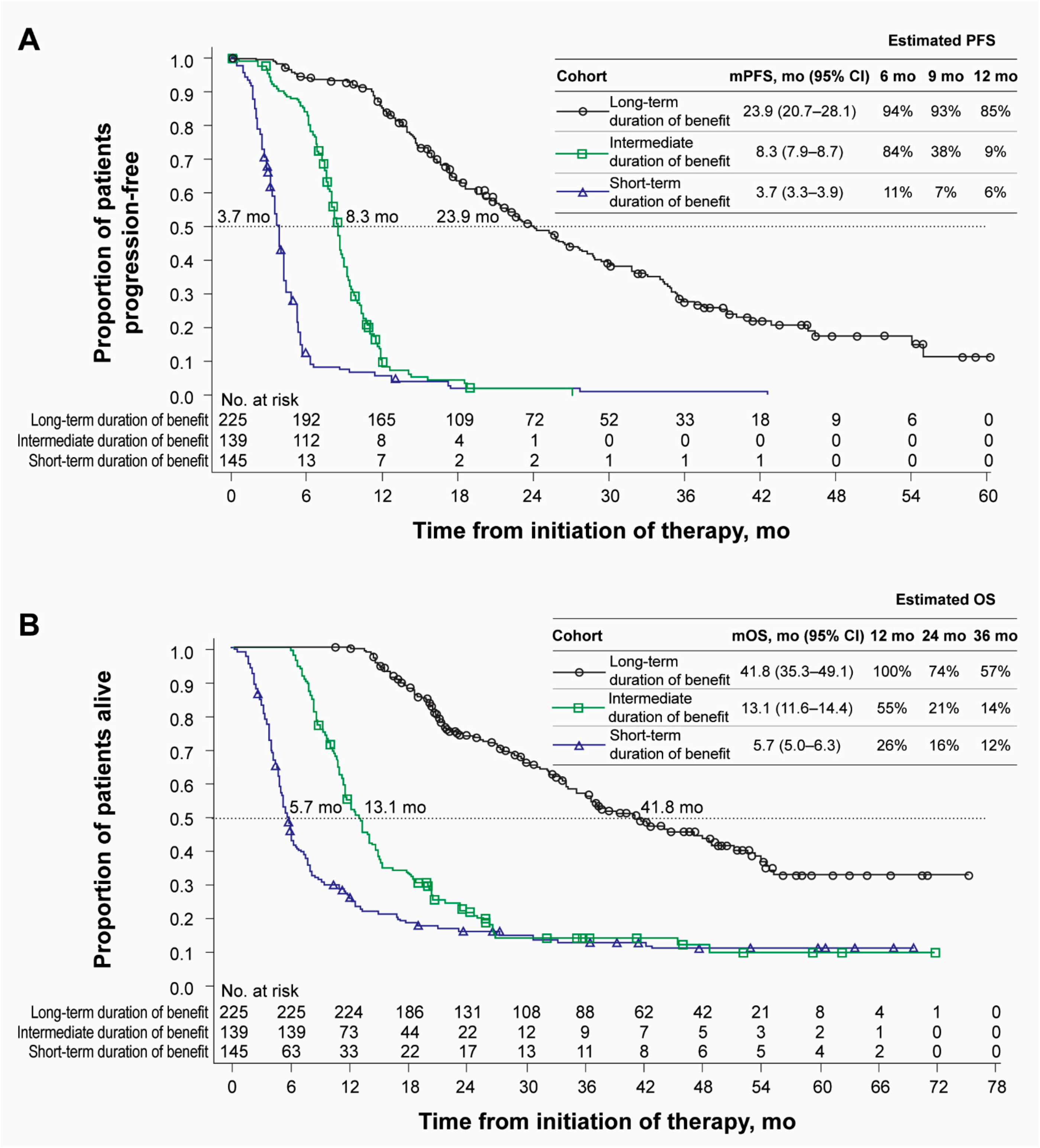


**Figure S1.** (**A**) PFS and (**B**) OS in patients in the long-term, intermediate, and short-term duration of benefit groups. CI, confidence interval; mOS, median overall survival; mPFS, median progression-free survival; OS, overall survival; PFS, progression-free survival.

**Table S1.** On-treatment deaths.

| **Primary reason for death, *n* (%)** | **Long-term Duration of Benefit, ≥12 months (*n* = 225)** | **Intermediate Duration of Benefit, ≥6 mo and <12 months (*n* = 139)** | **Short-term Duration of Benefit, <6 months (*n* = 145)** | **Overall (*N* = 509)** |
|---|---|---|---|---|
| On-treatment deaths | 32 (14.2) | 39 (28.1) | 56 (38.6) | 127 (25.0) |
| Melanoma | 24 (10.7) | 32 (23.0) | 49 (33.8) | 105 (20.6) |
| Other | 8 (3.6) | 7 (5.0) | 7 (4.8) | 22 (4.3) |
| Disease progression | 5 (2.2) | 4 (2.9) | 3 (2.1) | 12 (2.4) |
| Unknown | 1 (0.4) | 1 (0.7) | 0 | 2 (0.4) |
| Neoplasm progression | 0 | 1 (0.7) | 1 (0.7) | 2 (0.4) |
| Adverse event | 1 (0.4) | 0 | 0 | 1 (0.2) |
| Pneumonia | 1 (0.4) | 0 | 0 | 1 (0.2) |
| Pulmonary hypertension | 0 | 1 (0.7) | 0 | 1 (0.2) |
| Cerebral disorder | 0 | 0 | 1 (0.7) | 1 (0.2) |
| Pleural effusion | 0 | 0 | 1 (0.7) | 1 (0.2) |
| Salmonellosis | 0 | 0 | 1 (0.7) | 1 (0.2) |

**Table S2.** Reasons for dose reductions, interruptions, and permanent discontinuations.

| Reasons for dose reductions, interruptions, and permanent discontinuations | Long-term Duration of Benefit, ≥12 months ($n$ = 225) | Intermediate Duration of Benefit, ≥6 mo and <12 months ($n$ = 139) | Short-term Duration of Benefit, <6 months ($n$ = 145) | Overall ($N$ = 509) |
|---|---|---|---|---|
| Dose reduction of dab, n (%) | 34 (15.1) | 12 (8.6) | 9 (6.2) | 55 (10.8) |
| Reasons for dose reductions | | | | |
| Adverse event | 27 (79.4) | 8 (66.7) | 7 (77.8) | 42 (76.4) |
| Physician decision | 10 (29.4) | 6 (50.0) | 2 (22.2) | 18 (32.7) |
| Technical problems | 2 (5.9) | 0 | 0 | 2 (3.6) |
| Dose reduction of tram, n (%) | 19 (8.4) | 10 (7.2) | 2 (1.4) | 31 (6.1) |
| Reasons for dose reductions | | | | |
| Adverse event | 16 (84.2) | 6 (60.0) | 1 (50.0) | 23 (74.2) |
| Physician decision | 3 (15.8) | 4 (40.0) | 1 (50.0) | 8 (25.8) |
| Dosing error | 0 | 1 (10.0) | 0 | 1 (3.2) |
| Interruption of dab treatment, n (%) | 65 (28.9) | 35 (25.2) | 34 (23.4) | 134 (26.3) |
| Reasons for treatment interruptions | | | | |
| Adverse event | 61 (93.8) | 30 (85.7) | 28 (82.4) | 119 (88.8) |
| Physician decision | 11 (16.9) | 2 (5.7) | 0 | 13 (9.7) |
| Patient/guardian decision | 4 (6.2) | 1 (2.9) | 0 | 5 (3.7) |
| As per protocol | 2 (3.1) | 0 | 0 | 2 (1.5) |
| Disease improvement under study | 1 (1.5) | 1 (2.9) | 0 | 2 (1.5) |
| Interruption of tram treatment, n (%) | 57 (25.3) | 31 (22.3) | 25 (17.2) | 113 (22.2) |
| Reasons for treatment interruptions | | | | |
| Adverse event | 52 (91.2) | 25 (80.6) | 17 (68.0) | 94 (83.2) |
| Physician decision | 10 (17.5) | 3 (9.7) | 0 | 13 (11.5) |
| Patient/guardian decision | 3 (5.3) | 1 (3.2) | 0 | 4 (3.5) |
| As per protocol | 2 (3.5) | 0 | 0 | 2 (1.8) |
| Disease improvement under study | 1 (1.8) | 1 (3.2) | 0 | 2 (1.8) |
| Technical problems | 1 (1.8) | 0 | 0 | 1 (0.9) |
| Permanent discontinuation of dab, n (%) | 142 (63.1) | 134 (96.4) | 144 (99.3) | 420 (82.5) |
| Reasons for permanent discontinuations | | | | |
| Progressive disease | 105 (46.7) | 95 (68.3) | 105 (72.4) | 305 (59.9) |
| Death | 12 (5.3) | 16 (11.5) | 22 (15.2) | 50 (9.8) |
| Adverse event | 7 (3.1) | 14 (10.1) | 11 (7.6) | 32 (6.3) |
| Physician decision | 11 (4.9) | 3 (2.2) | 2 (1.4) | 16 (3.1) |
| Patient/guardian decision | 6 (2.7) | 2 (1.4) | 2 (1.4) | 10 (2.0) |
| Study terminated by sponsor | 0 | 3 (2.2) | 0 | 3 (0.6) |
| Completed study | 1 (0.4) | 1 (0.7) | 0 | 2 (0.4) |

| **Reasons for dose reductions, interruptions, and permanent discontinuations** | **Long-term Duration of Benefit, ≥12 months (*n* = 225)** | **Intermediate Duration of Benefit, ≥6 mo and <12 months (*n* = 139)** | **Short-term Duration of Benefit, <6 months (*n* = 145)** | **Overall (*N* = 509)** |
|---|---|---|---|---|
| Loss to follow-up | 0 | 0 | 2 (1.4) | 2 (0.4) |
| Permanent discontinuation of tram, n (%) | 135 (60.0) | 125 (89.9) | 124 (85.5) | 384 (75.4) |
| Reasons for permanent discontinuations | | | | |
| Progressive disease | 97 (43.1) | 86 (61.9) | 86 (59.3) | 269 (52.8) |
| Death | 12 (5.3) | 16 (11.5) | 22 (15.2) | 50 (9.8) |
| Adverse event | 9 (4.0) | 12 (8.6) | 11 (7.6) | 32 (6.3) |
| Physician decision | 9 (4.0) | 4 (2.9) | 1 (0.7) | 14 (2.8) |
| Patient/guardian decision | 6 (2.7) | 3 (2.2) | 2 (1.4) | 11 (2.2) |
| Loss to follow-up | 1 (0.4) | 0 | 2 (1.4) | 3 (0.6) |
| Study terminated by sponsor | 0 | 3 (2.2) | 0 | 3 (0.6) |
| Completed study | 1 (0.4) | 1 (0.7) | 0 | 2 (0.4) |

Abbreviations: dab, dabrafenib; tram, trametinib.
Note: A patient can have more than 1 dose reduction/treatment interruption; therefore, the percentages may sum to >100%.
